# Graphene Oxide: Study of Pore Size Distribution and Surface Chemistry Using Immersion Calorimetry

**DOI:** 10.3390/nano10081492

**Published:** 2020-07-30

**Authors:** Carlos A. Guerrero-Fajardo, Liliana Giraldo, Juan Carlos Moreno-Piraján

**Affiliations:** 1 Departamento de Química-Grupos de Investigación Aprena y Calorimetría, Facultad de Ciencias, Departamento de Química, Universidad Nacional de Colombia-sede Bogotá, Cra. 45 No. 26–85, Edificio 451, Bogotá 111321, Colombia; caguerrerofa@unal.edu.co (C.A.G.-F.); lgiraldogu@unal.edu.co (L.G.); 2 Facultad de Ciencias, Departamento de Química, Universidad de los Andes, Grupo de Investigación en Sólidos Porosos y Calorimetría, Bogotá 111711, Colombia

**Keywords:** graphene oxide, PSD, immersion calorimetry, hummer method, probe molecules, QSDFT, NLDFT

## Abstract

In this work, the textural parameters of graphene oxide (GO) and graphite (Gr) samples were determined. The non-local density functional theory (NLDFT) and quenched solid density functional theory (QSDFT) kernels were used to evaluate the pore size distribution (PSD) by modeling the pores as slit, cylinder and slit-cylinder. The PSD results were compared with the immersion enthalpies obtained using molecules with different kinetic diameter (between 0.272 nm and 1.50 nm). Determination of immersion enthalpy showed to track PSD for GO and graphite (Gr), which was used as a comparison solid. Additionally, the functional groups of Gr and GO were determined by the Boehm method. Donor number (DN) Gutmann was used as criteria to establish the relationship between the immersion enthalpy and the parameter of the probe molecules. It was found that according to the Gutmann DN the immersion enthalpy presented different values that were a function of the chemical groups of the materials. Finally, the experimental and modeling results were critically discussed.

## 1. Introduction

The materials area is one of the most studied in science today, due to its wide spectrum of applications. Some of the most widely investigated materials are those that have carbon as a fundamental element in their composition, which has various allotropic structures. Fullerene, carbon nanotubes, graphite (Gr), diamond, and graphene are part of these structures [1]. Graphene is the name given to a material that is characterized by having a monolayer of carbon atoms in its structure, packed in an arrangement similar to that of a honeycomb [2]. In this hexagonal arrangement, each carbon atom interacts by means of sp^2^ hybridization with three carbon atoms, originating a delocalized π bond that gives it exceptional properties [3]. Graphene is characterized by having different properties among which electrical, thermal, and optical can be mentioned. This material can be used in areas such as compounding for the electronic industry, thin films, the environment, catalysis, sensors, and biosensors, among others. From graphene, it is possible to obtain derivatives of it that have different chemical and physical properties useful in what refers to its applications [4,5,6]. One of these new materials is graphene oxide (GO), which contains abundant functional oxygen groups such as carboxyl, carbonyl, hydroxyl, and ether in the basal plane of the carbon sheet, which makes the GO intermediate layer hydrophilic and allows it to be subjected to intercalation processes, since it is possible that they are stacked in graphene oxide. GO can be used by taking advantage of its hydrophilic surface, which provides it with a favorable environment for the intercalation of polar organic molecules [7,8,9,10,11,12,13,14,15,16,17,18,19,20,21].

The interleaved GO is used in selective gas separation, an important area for its application and where there is still much to investigate. Establishing the characteristics of porous solids (Gr and GO) is significant to define the scope of the application area. Determining the surface chemistry and PSD (Pore Size Distribution) is part of the characterization of porous materials and the interaction of the solid with different molecules complements its knowledge [22]. To carry out the study of PSD with the data obtained from the gas isotherms at low temperatures, different methods are used which can be classified into two groups: classic methods and those with an integral approach of adsorption [22,23,24]. The classic methods are based on thermodynamic treatments, while the integral approach of adsorption is based on molecular modeling, mainly using the method of density functional theory (DFT) and statistical mechanics [22,25,26].

### Basic Fundamentals of PSD, NLDFT, and QSDFT (Pore Size Distribution, Non-Local Density Functional Theory and Quenched Solid Density Functional Theory)

The development of industrial applications of micro- and mesoporous materials requires obtaining a good characterization of the distribution of the porous structure, because with the pore size distribution (PSD) it will be possible to establish what the possible applications of such materials will be, for example in the gas storage and separation area, as well as its possible selectivity. One of the most popular and relatively simple methods for evaluating the PSD of different adsorbents is based on the analysis of the adsorption isotherms of nitrogen and argon at their respective boiling points. In summary, obtaining an accurate PSD for micro-meso porous materials is essential to establish the scope of their applicability. Unfortunately, obtaining and verifying the pore size distribution in this type of material is still a difficult task because the molecular arrangements that occur are highly disordered and very complex, in addition to the indirect techniques used that are also complex.

Experimentally, one of the current standards for microscopically determining PSD is to use indirect type molecular adsorption methods such as non-local density functional theory (NLDFT) and N_2_ isotherms at −196 °C. As determining PSD from NLDFT is an indirect method, validation may not be an easy task as it might seem for amorphous porous micro-meso materials, as reported in the scientific literature. This is important since it is known that this method can generate information about the surface from artificial calculations. Studies have been reported on the precision that can exist between the PSD calculated with the NLDFT and the exact geometric PSD, using different microporous materials, finding significant discrepancies between NLDFT and geometric PSD. On the other hand, other studies with these solids have found dominant peaks of NLDFT typically reported in the literature that do not necessarily represent the truly dominant pore size within the system. The confirmation provides concrete evidence of the results and variations arising from the use of the NLDFT method.

Although various methods can be used to determine the pore size distribution (e.g., small-angle X-ray scattering, mercury porosimetry, nuclear magnetic resonance, thermoporosimetry, and positron annihilation lifetime spectroscopy) [27,28,29], molecular adsorption [27,28,29,30,31,32] with the non-local density functional theory (NLDFT), is one of the most used methods today to determine PSD at the nanoscale.

Regarding the theories developed for PSD analysis, these are based on a reference adsorption isotherm of a nonporous solid, which is assumed to have the same chemical structure as that of the pore wall surface. Some theories such as the Barrett, Joyner, and Halenda method (BJH) [33], the modified version of BJH recently developed by Kruk, Jaroniec, and Sayari (KJS) [34,35], the theory of Broekhoff and Boer (BdB) [36,37], and its modifications [38,39,40,41] use the reference system directly in the form of an experimental t-curve. The Horvath–Kawazoe [42] (HK) method developed for microporous carbonaceous materials uses the Lennard–Jones (LJ) potential [42,43,44] derived from the perfect graphite sheets that make up the pore walls. The most sophisticated non-local density functional theory (NLDFT) [36,37,38,39,40,41,42,43,44,45,46,47] is also implicitly based on a reference adsorption isotherm of a nonporous solid because the solid-fluid molecular parameters are chosen to correlate the experimental reference isotherm. Therefore, the correct choice of a reference system is very important to obtain PSD reliably.

Regarding the NLDFT method itself, it uses a classical functional theory of fluid density to construct adsorption isotherms in ideal pore geometries (e.g., N_2_ adsorption in the slit-pore model at −196 °C). It is possible to obtain the result of the PSD, solving the integral adsorption equation (Equation (1), integral adsorption contribution, first term), which as mentioned by other authors [27,28,29,30,48,49,50,51,52,53,54], is a bad problem raised, using regularization techniques (in Equation (1), regularization contribution, second term) such as Tikhonov’s discrete regularization with non-negative least squares [27,34,35,36,37,38] or the numerical B-spline technique [27,48,49,50,51,52,53,54,55,56,57]. Therefore, some authors have proposed that the PSD solution be faced by introducing a parameter that will depend on the chosen regularization, λ (also known as smoothing parameter). In Equation (1), N_exp_ is the experimental adsorption of N_2_ at −196 °C, NLDFT represents the theoretical isotherms of N_2_ assuming an ideal pore geometry, such as slit and cylindrical pore, PSD is the pore size distribution, P/P_o_ is the pressure relation related to the saturation pressure of N_2_, and D is the pore diameter [27,57,58,59].

(1) $$N_{exp}\left(\frac{P}{P_{O}}\right)=\int_{D_{min}}^{D_{max}}N_{NLDFT}\left(\frac{P}{P_{O}},D\right)PSD\;\left(D\right)\;dD+\lambda \;\int_{D_{min}}^{D_{max}}{\left[{PSD}^{\prime \prime }\left(D\right)\right]}^{2}\;dD$$

The result of NLDFT PSD may also be different depending on the adsorption core chosen using various gases and assumed pore geometries [4,27]. Although the PSD obtained from NLDFT has provided valuable information on the characteristics of the materials, it can exhibit variations and some approximations such as artificial gap, which have been well-documented in the past for carbonaceous materials [27,48,49,50,51,52,53,54,55].

To minimize these variations that occur using the NLDFT PSD, several groups have worked and developed methods that explain energy and surface heterogeneity, such as 2D-NLDFT [27,32,33,34,35,36,47] and the quenched solid density functional theory (QSDFT) [47,48,49,50,51,52,53,54,55,56,57,58,59]. These methods have shown great promise in eliminating PSD variations and approximations within the specific cases studied for porous solids. Even though these improved models are available, recent literature still continues to report PSD with the simple pore slit model. This simple slit pore model is unlikely to be suitable for amorphous microporous materials that have highly heterogeneous surfaces and without well-defined pore geometry.

The evaluation of the PSD using the NLDFT kernel has shown that it is a reliable method for the characterization of certain materials, such as ordered silica, while it is difficult to analyze carbon materials. DFT methods were suggested for the study of the PSD of activated carbons [60,61,62,63,64], but the complexity regarding the heterogeneity of their porous structure has prompted the development of new characterization methods, which remains a current problem. As mentioned before, the NLDFT kernel for particularly carbonaceous materials is based on slit-independent pores with ideal graphite walls. This model has some drawbacks when applied, since when simulations are made from certain pore widths of more than a few molecular diameters, the modeled adsorption isotherms have some anomalies, such as stepped isotherms [60,65,66,67,68,69]. Experimentally, stepped adsorption isotherms are observed only at low temperatures for fluids adsorbed on smooth molecular surfaces, such as mica or graphite. However, in disordered materials, layered transitions are blocked due to the inherent geometric and energetic heterogeneities of real surfaces. 

There are several works to take into account the heterogeneity of carbon materials, such as molecular structural models using Monte Carlo inverse techniques [60,70,71,72], which are very useful, although very complex to be implemented for routine analysis of pore size. Computational simulations have been performed and they have shown that roughness and surface defects affect the shape of adsorption isotherms on heterogeneous surfaces [60,65,66,67,68,69,70,71,72]. Quenched solid density functional theory (QSDFT) [60] included this aspect for the various heterogeneous type materials with corrugated amorphous walls. QSDFT is a multi-component DFT, in which the solid is treated as one of the components of the adsorbate-adsorbent system. Unlike the conventional NLDFT kernel which assumes that the solid has graphical pore walls without structure, the QSDFT models the material using the distribution of solid atoms instead of the source of the external potential field. QSDFT allows to explicitly explain the effects of surface heterogeneity [60]. The heterogeneity of the surface in the QSDFT model is characterized by a single roughness parameter that represents the characteristic scale of the surface undulation. The density functional theory implies that, under the conditions of thermodynamic equilibrium, the spatial distribution of the adsorbed species corresponds to the minimum of the grand thermodynamic potential in given chemical potential (s), pore volume and temperature. The QSDFT model [60,64,65,66,67,68,69,70,71,72] is based on the multicomponent density functional theory method, in which the grand thermodynamic potential Ω is defined as (Equation (2)) [60]:

(2) $$\Omega \;\left[\left\{\rho_{i}\left(r\right)\right\}\right]=F_{int}\left[\left\{\rho_{i}\left(r\right)\right\}\right]+\underset{i}{\sum }\int dr\rho_{i}\left(r\right)\left[\psi \left(r\right)-\mu_{i}\right]$$

where *F_int_* is Helmholtz’s intrinsic free energy, *q_i_* and *μ_i_* are the local numerical density, and chemical potential of component *i*, and *Ψ*_i_ are the local external potentials.

In the QSDFT single-component adsorption model, we consider the solid as a quenched component of the two-component solid-fluid system. Adsorption interactions are reduced to pairwise interactions between adsorbate (fluid) and adsorbent (solid) molecules, and external potentials are not considered. Therefore, the grand thermodynamic potential is reduced to (see Equation (3)):

(3) $$\Omega \;\left[\rho_{s}\left(r\right);\rho_{f}\left(r\right)\right]=F_{int}\left[\rho_{s}\left(r\right);\rho_{f}\left(r\right)\right]-\mu_{s}\int dr\rho_{s}\left(r\right)-\mu_{f}*\int dr\rho_{f}\left(r\right)$$

Minimization of the large thermodynamic potential is done with respect to the fluid density *ρ_f_*(*r*) by maintaining the solid density *ρ_s_*(*r*) and the contributions form unchanged solid-solid atomic interactions. The complete development is widely described in the literature.

For determining PSD, a number of questions that need to be solved arise and have been recently discussed in the scientific literature [22]. These aspects are related to the uncertainty of the PSD according to the method used and knowing if there are differences between these methods [22]. Various studies with activated carbons and different gases have been carried out to analyze these effects in detail [22,23,24,25,26,27] to establish those of the main sources of uncertainty in the calculations of the PSD evaluated using the integral approach of adsorption [22,73,74,75,76,77,78,79,80,81,82,83,84]. Several authors carry out an analysis of these aspects, making interesting contributions to the calculation of the PSD from the results of gas isotherms on porous solids [22,68,69,70,71,72,73,74,75,76,77,78,79,80,81,82,83,84].

It is known that when placing a solid in contact with a liquid or a vapor, due to physical or chemical interactions, thermal effects are generated, which can be quantified by different methods. One of the experimental techniques used to determine these interactions is immersion calorimetry that allows measuring the thermal effects generated by the interactions (in this study: solid-liquid) that occur between a solid and a vapor or a solid and a liquid, directly and without making approaches in the methodology. Immersion calorimetry leads to the characterization of the surface properties of adsorbents, including activated carbons, carbon xerogels, carbon aerogels, among others, and evaluating properties such as specific surface area [22,47,48,49,50,84]. Some results were recently published in which the immersion calorimetry leads to evaluation of not only the surface areas but also the size distributions of the micropores of the activated carbons.

The enthalpy of immersion of solids in liquids of different sizes allows establishing the intensity of the interaction of the solid with each molecule and involves its ability to enter the pores of the solid. There are some works that relate immersion enthalpy to pore diameter but regularly there are no published works in the scientific literature with studies of immersion enthalpy for nanomaterials, specifically graphite and graphene oxide. Therefore, the scope of this research is to explore the relationship between the immersion enthalpy of Gr and GO in different probe molecules that have a broad spectrum with respect to their molecular kinetic diameter and compare them with the PSD calculated with the kernels NLDFT and QSDFT. Therefore, the scope of this research is to explore the relationship between the immersion enthalpy of Gr and GO in different probe molecules that have a broad spectrum with respect to their molecular kinetic diameter and compare them with the PSD calculated with the kernels NLDFT and QSDFT. For this, N_2_ isotherms were determined at −196 °C for Gr and GO synthesized by our research group using the Hummer method, the PSD were evaluated using the NLDFT and QSDFT models and compared with the immersion enthalpy of the adsorbents in each of the 15 probe molecules to cover a broader range of molecular size (0.30 nm to 1.50 nm) [22,72,84,85]. Additionally, this research studies the possible correlations between the surface chemistry of the solids, the enthalpy of immersion, besides the correlations with Gutmann DN, the dielectric constants and the dipole moments of the respective molecules.

## 2. Materials and Methods 

### 2.1. Materials and Reagents

Table 1 summarizes the characteristics of the reagents used in this work. The molecules in which the immersion enthalpy of Gr and GO will be determined have been selected taking into account their kinetic diameter with the purpose of relating the ΔH_imm_ with the PSD obtained from the N_2_ adsorption isotherm. Fifteen (15) probe molecules (PM) with different molecular sizes were chosen for their study with the solids and whose purity, manufacturer’s trademark, and the acidity constant expressed as pK_a_, are shown in Table 1.

Table 1 reports the initial purity given by the supplier of the chemical reagent and thus the reagent is used in the determination of enthalpy; a later purity is not provided, because the PM are used in the same condition that they arrived to the laboratory, since they have a purity suitable for immersion calorimetry experiments. Once each test has been carried out, the reagent is removed together with the adsorbent and the solvent is treated according to the safety regulations to be disposed. No purification procedure is carried out.

### 2.2. Characteristics of the Samples Used in this Work

GO was prepared in our laboratory [86] from graphite sheets (Flakes, Sigma-Aldrich, St. Louis, MO, USA, CAS 7782-42-5) using the improved Hummers method reported in the literature and taking into account the changes suggested by several authors to carry out a procedure with good performance and laboratory safety [86]. A brief summary of the procedure carried out is as follows: a main process consisting of oxidation. KMnO_4_ (Sigma-Aldrich, CAS 10294-64-1) which is added in several portions, maintaining a 9:1 ratio of H_2_SO_4_/H_3_PO_4_ (360:40 mL) (concentrated acids, Sigma-Aldrich CAS 7664-93-9 (H_2_SO_4_) and Sigma-Aldrich CAS 7697-37-3 (HNO_3_)) previously prepared, and graphite flakes (3.5 g, 1.17 equiv.), in this step the temperature was controlled so that it did not exceed the range of 35 to 40 °C. After heating the system to 50 °C and stirring for 36 h, it was left for 24 h, until the mixture cooled naturally. With a cryostat, the temperature of the mixture was lowered to 1 °C. Subsequently, H_2_O_2_ (30%, Sigma-Aldrich CAS 7722-84-1) was added dropwise to remove excess KMnO_4_. The mixture was subsequently adjusted to pH 1 by the addition of deionized water (3.0 × 10^−7^ ohm^−1^·cm^−1^, Lab Manager™). Then, the solution produced was centrifuged (Universal Z 326K refrigerated centrifuge, Hermle TMBrand, Wehingen, Germany) at 7000 rpm for 20 min until the solid separated from the liquid. The precipitate was then washed with deionized water, HCl (36.5–38%, Sigma-Aldrich, CAS 7647-01-0) and ethanol (99.5%, Sigma Aldrich) consecutively, five times, and then coagulated with diethyl ether (99%, Sigma-Aldrich, CAS 60-29-7). This latter solvent was removed by carefully heating the solution at 45 °C. GO was then peeled off with ethanol using an ultrasound bath (Fisher Brand, Model CPXH, Boston, MA, USA) for 90 min. Finally, the GO was dried at 80 °C for 24 h and ground in an agate mortar, until it passed through a 100 mesh [86].

### 2.3. N_2_ Adsorption Isotherms at −196 °C

To determine the textural characteristics of both graphite (Gr) and graphene oxide (GO), nitrogen adsorption-desorption isotherms were performed at −196 °C using an IQ2 sortometer (Quanthachrome Inc, Boyton Beach, FL, USA). The protocol used to carry out the measurements and ensure good reproducibility in the data consisted initially degassing the samples at a temperature of 250 °C, applying a high vacuum (10^−5^ mbar) for 5 h, a procedure that ensures eliminate all those pre-adsorbed species that could affect the measurements. The Brunauer-Emmett-Teller (BET) model [85,86,87,88] was used to evaluate the specific surface area taking the data in the range at a P/P^o^ between 0.05–0.35, which presents a good linearity. On the other hand, the Dubinin-Radushkevich models (DR) [89,90,91,92,93] and the functional theory of density (DFT) [94] were used to complement the studies of the textural properties of the solids under study. Then, by means of the DR equation the volume of micropores DR (V_o_) was calculated, while the average pore width, L_o_, was evaluated using the following expression [89]: L_o_ = 10.8/(Eo − 11.4), where E_o_ is the energy characteristic obtained by applying the DR equation to experimental data. The equation was applied in the relative pressure range 2 × 10^−6^ ≤ P/P^o^ ≤ 0.2. The pore size distribution (PSD) was calculated using the methods corresponding to the theories of non-local density functional theory (NLDFT) and the quenched solid density functional theory (QSDFT) assuming cylindrical, slit, and combined pore models of cylindrical-slit geometries for the pores of the solids under study (Gr and GO) [89,90,91,92,93,94,95,96].

### 2.4. Determination of Functional Groups Using Boehm Titrations

Boehm titration [97,98] was performed to determine the functional surface groups of Gr and GO. Solutions of HCl (0.05 mol L^−1^), NaOH (0.05 mol L^−1^), NaHCO_3_ (0.05 mol L^−1^), and Na_2_CO_3_ (0.1 mol L^−1^) were prepared. The NaOH solution was standardized by KHC_8_H_4_O_4_ and the HCl solution was standardized by Na_2_CO_3_ solution. Both Na_2_CO_3_ and NaHCO_3_ were oven dried at 110 °C for 6 h before standardizations were carried out. The solids were pre-dried at 110 °C for 3 h. 10 mL of each solution were added to 0.05 g of each solid in a bottle of 25 mL and placed on a stirrer for 24 h. The mixture was filtered. From the filtrate, 2.0 mL was taken and titrated using standard NaOH for the solution that contained HCl and standard HCl was used to titrate the basic solutions (NaOH, NaHCO_3,_ and Na_2_CO_3_). For the titration of the HCl and NaOH solutions, phenolphthalein was used as an indicator, while for Na_2_CO_3_ and NaHCO_3_, the pH meter was used. The basic content of samples was calculated from the amount of HCl that reacted with the solids. Acid groups were calculated using the Boehm criteria as follows: (a) NaOH neutralizes carboxylic, phenolic, and lactonic groups; (b) Na_2_CO_3_ neutralizes the carboxylic and lactonic groups; and (c) NaHCO_3_ neutralizes only the carboxylic group. The results presented in this work correspond to the average of a triplicate. Experiments were carried out to carry out the adjustments to remove CO_2_ as suggested by Goertzen et al. [98]. A pH meter was used. The basic content of the group of samples was calculated from the amount of HCl that reacted. Acid groups were calculated using the Boehm criteria as follows: (a) NaOH neutralizes carboxylic, phenolic, and lactonic groups; (b) Na_2_CO_3_ neutralizes the carboxylic and lactonic groups; and (c) NaHCO_3_ neutralizes only the carboxylic group. The results presented in this work correspond to the mean of a triplicate. Experiments were carried out to make adjustments to remove CO_2_ as Goertzen et al. [98] suggested. 

### 2.5. Immersion Enthalpy Studies

#### 2.5.1. Description of Immersion Calorimeter

Calorimetric measurements were performed on a “home-made” immersion calorimeter developed in our laboratory [99,100,101]. Calorimetry is a technique that needs to be carried out carefully to obtain reliable measurements and reproducible experiments; for this reason, when carrying out each immersion calorimetric experiment, the different effects generated during the measurement must be taken into account and quantified, in addition to the one desired to be measured, that is, the net solid-liquid interaction. Therefore, the heat that is recorded in an immersion experiment is the sum of different contributions as shown in the following expression [99]:

(4) $$Q_{exp}=\Delta_{imm}U+W_{b}+\overset{V-v}{\underset{0}{\int }}pdv+\frac{\Delta_{hq}}{RT}\left[\left(p-p^{o}\right)V+p^{o}v\right]$$

where: $\Delta_{imm}U$ is the immersion Energy, $W_{b}$ is the work of breaking vial, $\int_{0}^{V-v}pdv$ is work of vapor compression in the bulb, $\frac{\Delta_{hq}}{RT}\left[\left(p-p^{o}\right)V+p^{o}v\right]$ is liquefaction of the liquid in the bulb (*v*), and liquid vaporization outside the bulb (*V*).

The interesting thing about Equation (1) is that each one of the terms described there can be evaluated by performing “white” experiments, using for them glass bulbs of different sizes and calculating the average thermal effect generated by the breakage of these [99,100]. This aspect is important to highlight since not taking it into account generates uncertainty in the results they present, which prevents the possibility of examining the true scope of this methodology. The equipment used in this work has been described in the scientific literature [99,100,101]. It consisted of a 10 mL stainless steel cell, specially designed so that the glass bulb broke easily with a slight impact, so that the solid came into contact with the immersion liquid quickly and easily. The calorimeter had an electrical resistance, that allowed to carry out the respective electrical calibration, which permitted to verify every time that the equipment was working correctly and a device in which the glass bulb was fixed (which had a shape, size, and thickness capable of guaranteeing reproducibility in the thermal effect generated during the break). Before doing each calorimetric experiment, the sample inside the bulb was a vacuum (10^−5^ mbar and at a temperature of 373 K) with the sample previously weighed.

**Figure 1:**
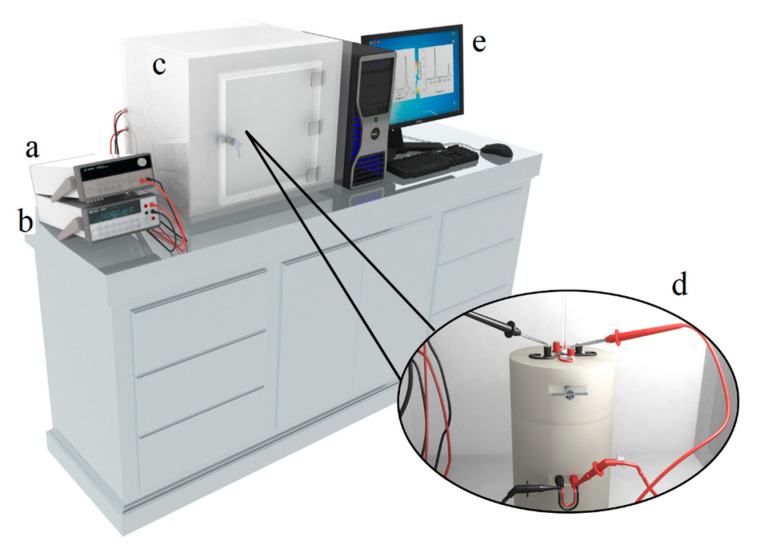
Immersion calorimeter design. The diagram shows: (**a**) the multimeter, (**b**) amperimeter, (**c**) compartment where the calorimeter is located isolated from the surroundings, (**d**) a zoom that shows the calorimeter, (**e**) computer that registers the calorimetric signals.

The immersion calorimeter is shown in Figure 1; it was then assembled inside an aluminum block so that the thermal energy generated in the solid-liquid interaction was quickly conducted to the surroundings, being registered by the sensors of the thermal effect. These thermoelectric modules (Tellurex brand™, Traverse City, MI, United States) were the semiconductive type and four were arranged in series around the calorimeter cell. These thermoelectric modules generated a voltage signal due to the Seebeck effect, which was proportional to the heat flow from the calorimeter cell. The thermoelectric potential was measured with an Agilent 6^1/2^ digit multimeter, model 34349, connected to a computer through an RS−232 interface. To stabilize the signal, the calorimeter was placed inside a block made of Teflon (Figure 1) [99,101].

#### 2.5.2. Immersion Calorimetry Experimental

In a typical immersion calorimetry experiment, 0.1 g of Gr or GO are weighed and placed in the glass bulb (higher weights were used for some liquids in this work). The sample is then desorbed at a temperature of 373 K and a vacuum at 10^−5^ mbar for 3 h. Subsequently the glass bulb is sealed and placed in the calorimeter, simultaneously 10 mL of the immersion liquid is placed in the calorimeter cell.

The signal stabilized until a baseline around ± 0.50 μV was achieved. At that moment, the glass bulb was broken and the thermal effect of the interaction was recorded; the signal was allowed to reach the baseline again. Then, the electrical calibration was carried out. Finally, corrections for glass bulb breakage and liquid evaporation within the bulb were made by repeating experiments with empty bulbs. Three experimental determinations were made for each liquid with the Gr and GO for each sample and the average values of the immersion enthalpy were considered. The probe molecules used in this work are shown in Table 1. 

Additionally, taking advantage of the versatility of immersion calorimetry, this technique was used to investigate the possible relationships between immersion enthalpies in the different probe molecules and the possible presence of acid and basic groups present in the Gr and GO. A wide spectrum of molecules with different properties that differ in electron donor properties in addition to their molecular diameter were used within the probe molecules. These molecules are reported in Table 1. The parameter that represented the electron donor character of the solvent is the Gutmann Donor Number (DN) [102], which corresponded to the measure of the ability of a solvent to solvate cations (acids Lewis) due to its ability to act as a donor of electron pairs. Gutmann DN was expressed in kcal mol^−1^ and it was related to other properties better known as dielectric constant and dipole moment.

## 3. Results and Discussion

### 3.1. Analysis of N_2_ Adsorption Isotherms at −196 °C 

**Figure 2:**
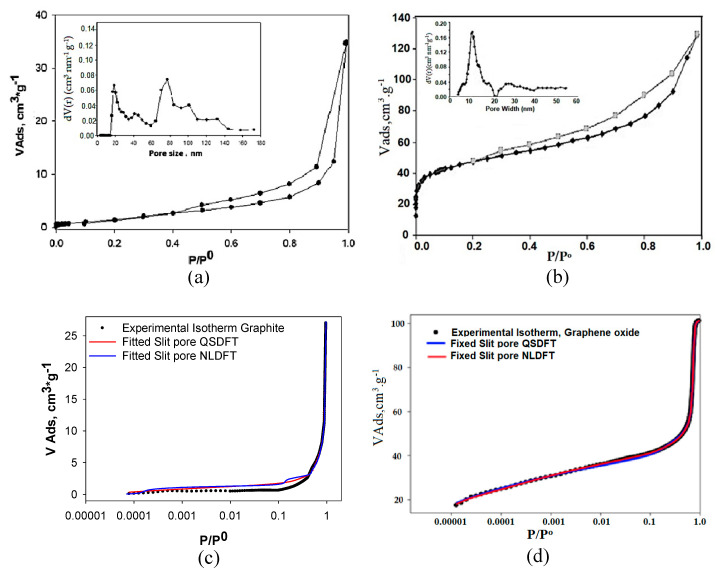
N_2_ gas adsorption-desorption isotherms of samples and pore size distribution (PSD) calculated with quenched solid density functional theory (QSDFT) kernel: (**a**) graphite (Gr) (**b**) graphene oxide (GO) (**c**) Simulation of Gr with non-local density functional theory (NLDFT) and QSDFT (**d**) Simulation of GO with NLDFT and QSDFT [86].

Figure 2 and Table 2 show the adsorption isotherms of N_2_ at −196 °C and the parameters obtained for the corresponding textural characteristics for graphite (Gr) and graphene oxide (GO) which were previously analyzed [86]. The results show the structural differences between the starting material Gr and the GO synthesized later. The Gr isotherm showed a Type IV isotherm with a H4 type hysteresis loop; the loop was between 0.4 and 0.99 of P/P^o^. While the GO presented a combination of I and IV isotherm type and a hysteresis loop at a P/P^o^ between 0.25 and 0.95. The type of isotherms and the type of pore were assigned according to the IUPAC (International Union of Pure and Applied Chemistry) classification [103]. The specific BET area for graphene oxide was 47.5 m^2^ g^−1^, which was greater than the one for Gr. This showed that during the GO synthesis procedure from Gr, the textural part of this reagent was modified during the synthesis process towards GO, and due to the treatment chemical used and subsequent exfoliation to which the Gr was subjected, the area was increased. On the other hand, the DR model was used to evaluate the micropore volume (V_micr_, cm^3^ g^−1^), the characteristic energy of adsorption (E_o_, kJ mol^−1^), and pore radius (Å), obtaining as results for the GO the following values: 0.154, 18.60, 9.3, respectively; those are found in Table 2. In order to evaluate the microporosity, it is necessary to start from the fact that the dimensions of these structures are of the molecular order, which can rule out the possibility of capillary condensation, in addition to the amount of gas adsorbed in saturation conditions, expressed as a liquid, it provides a measure of the pore volume of the solid (Gurvitsch rule). This filling occurs in the form of a liquid due to the adsorption potential of the micropores. However, most microporous adsorbents have appreciable external surfaces and larger pores (mesopores), which does not allow the Gurvitsch rule to be applied in a direct way to determine the volume of micropores. Another method that has been successfully applied to evaluate the volume of micropores in solids with a high percentage of microporosity is the Dubinin-Radushkevich (DR) model, which is based on the Polanyi potential theory. The linear form of the Dubinin-Raduskevich equation is:

(5) $$\log V={\mathrm{logV}}_{0}-\;D\;{\left(\log\frac{p^{0}}{p}\right)}^{2}$$

(6) $$D=0.43\;B\frac{T^{2}}{\beta^{2}}\;B=2.303^{2}R^{2}K$$

where D is a constant related to the characteristic energy of adsorption and the average pore size of the solid, β is the Dublin constant of affinity coefficient, K is a constant that characterizes the Gaussian distribution of pore size and V_o_ is the volume of micropore. Representing log V against log^2^ (P/P^o^), a linear relationship will be obtained as long as the size of the micropores has a uniform Gaussian distribution and whose intercept on the x axis will be the value Log Vo and therefore the volume of micropores is obtained. The validity range of the Dubinin-Raduskevich equation ranges from P/P^o^ = 10^−5^ to 0.2–0.4. This range is established because at lower pressures of 10^−5^ the filling of smaller micropores or ultra-micropores is carried out and at pressures above 0.4 the filling of the mesopores begins, processes that are not contemplated in the model theory. Although the DR model has been widely used in the characterization of porous solids, it has some disadvantages; how not to consider the effect of pore size and shape on molecular packaging. This problem has been addressed in the methods based on molecular simulation (MC) and in the theory of functional density (density functional theory, DFT). In this investigation the model DFT (density functional theory) was used for the determination of the pore volume distribution (also for GO-besides of Gr-) (V_p_, [cm^3^ g^−1^]) and the pore size and half pore width [nm] (0.18 and 8.75 respectively). The results of these variables for GO compared with those obtained for Gr, clearly showed that a textural change occurred to this material until it became GO, as it was shown in a previously published study of ours [88]. The inner box shows the pore size distribution (PSD) calculated using the QSDFT kernel (quenched solid density functional density) for both Gr and GO. Only the results of distributions obtained using this kernel were presented because they showed the best fit with respect to the experimental data, as it can be seen in Table 3. Modeling was performed for both Gr and GO assuming slit, cylinder (cyl), and combined (slit-cyl) pores. The lowest percentage of error (%) for both samples was obtained when the cylindrical pore (cyl) (4.364% Gr; 1.354% GO) was modeled using the QSDFT kernel. This was coherent if it was taken into account that the structures that had not only the Gr but the GO, they were of a “graphitic” nature—they were samples that could be modeled as laminar, resembling a “slit”. When the pore widths for Gr and GO were calculated using the NLDFT and QSDFT kernels they differed slightly, but being consistent with the type of isotherm that each material presented, especially GO.

The results presented so far showed that the average pore width calculated by QSDFT (quenched solid density functional theory) indicated small pore values, around 7.80 nm to 8.75 nm for Gr and GO respectively, which confirmed the structural change of Gr as starting component to obtain GO. This is associated with this is the chemical process by which was synthesized graphene oxide; during the process from graphite to graphene oxide, chemical reagents enter the graphite layers, exfoliating them and additionally altering their surface chemistry. This is what is shown performing the analysis with kernels NLDFT and QSDFT.

Using the IQ2 autosorb analyzer software, the simulated isotherms were run using the NLDFT and QSDFT kernels for Gr and GO. The results are shown in Figure 2c,d. The blue lines show the simulated isotherms for the NLDFT kernel and the red lines for the QSDFT kernel. As observed, the experimental points were better adjusted to the simulated isotherm with the QSDFT kernel. This, as mentioned in the materials and methods section, led to a better fit due to the fact that in its thermodynamic foundation it was taken into account the surface roughness of the solid. This result was consistent with what had been analyzed so far.

### 3.2. Analysis of the Results of the Chemical Groups: Boehm Titrations and Immersion Calorimetry

Table 4 shows the results obtained for the evaluation of functional groups using the Boehm method. These showed that for Gr it was not possible to register chemical groups associated with the surface according to the titrations that were carried out with the bases of different strength, which allowed inferring that the method did not permit the detection of functional groups that were neither acidic nor basic. On the other hand, for GO, different functional groups were determined, which are reported in Table 4; these functional groups were those that the literature has reported as those that are normally present in GO. Additionally, a greater amount of total acidity than total basicity was obtained, which was consistent with what has been found by the specialized literature, if the pH_pzc_ of GO was taken into account [86].

The immersion enthalpy for Gr and GO in HCl and NaOH was determined. Initially it is necessary to mention that the registered thermal effect for the Gr corresponded to the interaction between the water molecules and the graphite sheets. Blanks were made and the results obtained corresponded to the net immersion heat generated for this interaction; the value obtained for Gr for both HCl and NaOH was around 10 J g^−1^; similar to the value obtained for the immersion of Gr in water. On the other hand, the immersion enthalpy obtained for the GO immersed in HCl and NaOH was 30.43 ± 0.03 J g^−1^ and 48.57 ± 0.03 J g^−1^ respectively. This was very consistent with the results obtained by Boehm titrations, where it was assumed that NaOH neutralized carboxylic, lactone and phenolic groups, Na_2_CO_3_ carboxylic and lactone groups, while NaHCO_3_ only reacted with carboxylic groups. According to that, GO had a greater amount of acid groups with respect to the basic ones, as mentioned before. When comparing these results with those found with immersion calorimetry, it was observed that they were consistent.

**Figure 3:**
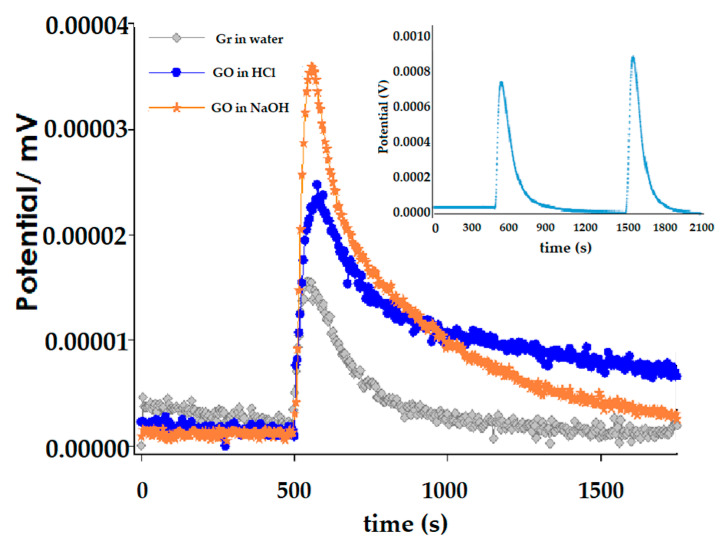
Immersion thermograms of Gr and GO: (**gray line**) Gr in water, (**blue line**) GO in HCl and (**orange line**) GO in NaOH. In the internal box a typical thermogram.

Figure 3 shows the characteristic results for a test of the immersion of Gr and GO in HCl and NaOH. In the inner box of this figure is presented the typical thermogram that was generated in an immersion calorimetry experiment that is obtained in each measurement: the first peak corresponded to the immersion of GO in the adsorbate and the second peak corresponded to the electrical calibration. Figure 3 clearly shows that a greater thermal effect occurred by immersing GO in NaOH than in HCl; this indicates, as mentioned above, that the effect was correlated with the surface groups determined by Boehm’s method, where in this case the thermal effect corresponded to the reaction of NaOH with the acidic groups that were the ones in greatest quantity and also the accessibility of NaOH to them. The peak was lower in the case of immersion of GO in HCl, where the effect showed the neutralization of groups of a basic nature. This is in good agreement with the functional groups determined by Boehm titrations, which are presented in Table 4. In the next part, the immersion enthalpies of each of the probe molecules will be analyzed in both Gr and GO. It is worth mentioning that due to the wide spectrum of characteristics that these molecules possess (as can be seen in both Table 1 and Table 5), different types of solid-liquid interactions are registered. Probe molecules possess not only different kinetic diameter but different pK_a_. Then, some of these molecules will not have chemical interactions with solids, such as benzene, cyclohexane, and others will have different types of interaction according to the order of magnitude of the pK_a_ and the functional groups, particularly for GO.

### 3.3. Immersion Calorimetry Results Analysis Using Different Probes Molecules

**Figure 4:**
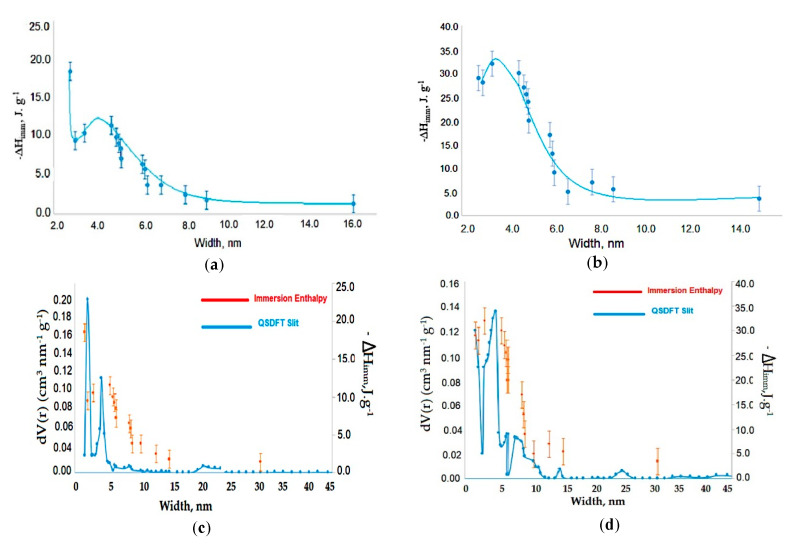
Immersion enthalpy of the probe molecules. (**a**) Immersion enthalpy as a function of kinetic diameter for Gr. (**b**) Immersion enthalpy as a function of kinetic diameter for GO. (**c**) Immersion enthalpies and pore distribution calculated using the QSDFT (slit pore geometry) as a function of the kinetic diameter of the probe molecules for Gr. (**d**) Immersion enthalpies and pore distribution calculated using the QSDFT (slit pore geometry) as a function of the kinetic diameter of the probe molecules for GO.

To analyze the results of the immersion enthalpy obtained with the probe molecules based on the pore size distribution (PSD), only the area corresponding to the porous micro-meso was considered. Figure 4 shows the results obtained for immersion enthalpy for both Gr and GO, as a function of the kinetic diameters of the probe molecules used in this study. As can be seen, the results are adjusted to “polynomial” functions for both Gr and GO, which have a characteristic and a particular tendency for each sample. In Figure 4a, corresponding to the Gr, and the enthalpic values for this curve tended to center towards values of molecules with a pore diameter of 4 nm; looking in detail at Figure 4a, between 5–6 nm, the enthalpy values rose and fell describing a “second peak” which was not shown in the graph because the data had been “smoothed” with the polynomial function. This may allow thinking about a possible “molecular sieve” effect on the diffusion of the probe molecules inside the Gr pores system. On the other hand, the curve of the graph shown in Figure 4b (corresponding to the GO) is centered towards the probe molecules with a diameter of 3.5–5 nm. The results show that the entrance to the porous system of both Gr and GO of each of the probe molecules, depends on the pore size and on the molecule, itself. In the case of Gr, the generation of higher immersion enthalpy values is found for the molecules with a lower kinetic diameter, while the others do not fully penetrate and tend to generate a “molecular sieve-like” effect because they do not achieve penetration of the pores; on the other hand, for the GO sample, since it has a slightly broader pore diameter, in addition to the presence of functional groups, this generates a wide spectrum of immersion heats related to the diffusion of molecules into the porous system and their interaction with functional groups.

The wide versatility of immersion calorimetry, which is related to solid-liquid interactions, in this particular case to those that are functions of the area and the surface chemistry, allows that the thermal effect be generated and recorded. This research analyzes and correlates this generated energy, depending on the properties that were mentioned. Some of the probe molecules used in this investigation may possibly establish specific interactions with the surface due to their chemical nature; in this case the energy generated in each experiment is directly related to the intensity of the interaction. For example, if the hydrophilic nature of GO is taken into account, and the abundance of –C–O–C– and -OH groups on its surface, in addition to C=O and COOH groups at the edges, it can only be dispersed in aqueous media. So, it is important to have clarity of the thermal effect that is registered in the systems under investigation: each experiment records the net thermal effect generated when the solid is placed in contact with the probe molecule, the breakage of the vial and the interactions between the solid-probe molecule, which in some may include delamination. These are the effects that are reported and correlated in this work.

On the other hand, the comparison between the results of the immersion enthalpies into the probe molecules used in this investigation and the PSD that were obtained for the micro-meso zone using the QSDFT kernel (performing a modeling for the slit-type pores, the ones with the best fit as mentioned before) for both Gr and GO was presented in Figure 4c,d, respectively. These results were very interesting, consistent and could be associated with what was found and analyzed in Figure 4a,b for Gr and GO, respectively. Figure 4c corresponding to Gr showed the PDS calculated with the QSDFT kernel and a third axis had been included, in which the immersion enthalpies obtained with the probe molecules were presented, related to the range of their kinetic diameters. When the results presented in Figure 4c are analyzed, it can be seen that the immersion enthalpies tend to perfectly describe the same PSD calculated with the QSDFT kernel; It is a really interesting and new result, which shows that immersion calorimetry as a function of the diameter of the probe molecules, allows to track the PSD for this type of solids. On the other hand, an interesting result is also observed when comparing the results of Figure 4b,d: Figure 4b presented a single peak between 4–5 nm as a function of immersion enthalpies, a result that is in good agreement with Figure 4d, which has a pronounced dominant peak around 4–5 nm.

In this case, the kernel showed a clear peak centered towards 5 nm, which coincided with what the immersion calorimetry tests showed.

In summary, the benefits of this approach are important and novel: no assumptions needed to be made for the shape of the probe molecule or the adsorbent pore geometry; geometric defects within the pores and connectivity between pores were included in the measured enthalpy. Experiments were typically carried out at room temperature and controlled, thus avoiding the diffusion restrictions of the probe molecule and no adsorbed equilibrium of the absorbent is necessary. Of course, contributions from high energy sites on the surface would lead to specific interactions if the probe molecule also contained a large dipole moment and/or functional groups and the resulting energy released would suggest a higher surface area than the one actually available [22,23,24]. Polar surface sites and their possible contributions can be explored separately by comparing the immersion energy for benzene with that of pyridine. Their similar sizes probed the same pore dimensions, while the latter also took into account the contributions of the (relatively acidic) surface functional group. According to what was described here, immersion calorimetry can be used as a model-independent technique for the determination of PSD of materials that are fundamentally microporous. A systematic study such as the one carried out in this research, a set of liquids with known increasing kinetic diameters ranging from 0.33 nm (dichloromethane) to 1.50 nm (Tri-2,4-xylyphosphate) were taken, with a reasonable interval between the diameter of the chosen probe molecules, which generated a PSD based on the molecular sieve principle. Each measured immersion enthalpy could either be used directly or converted to available surface area for PSD determination. Therefore, it did not require modeling to reduce the data in a PSD [22,23,24].

**Figure 5:**
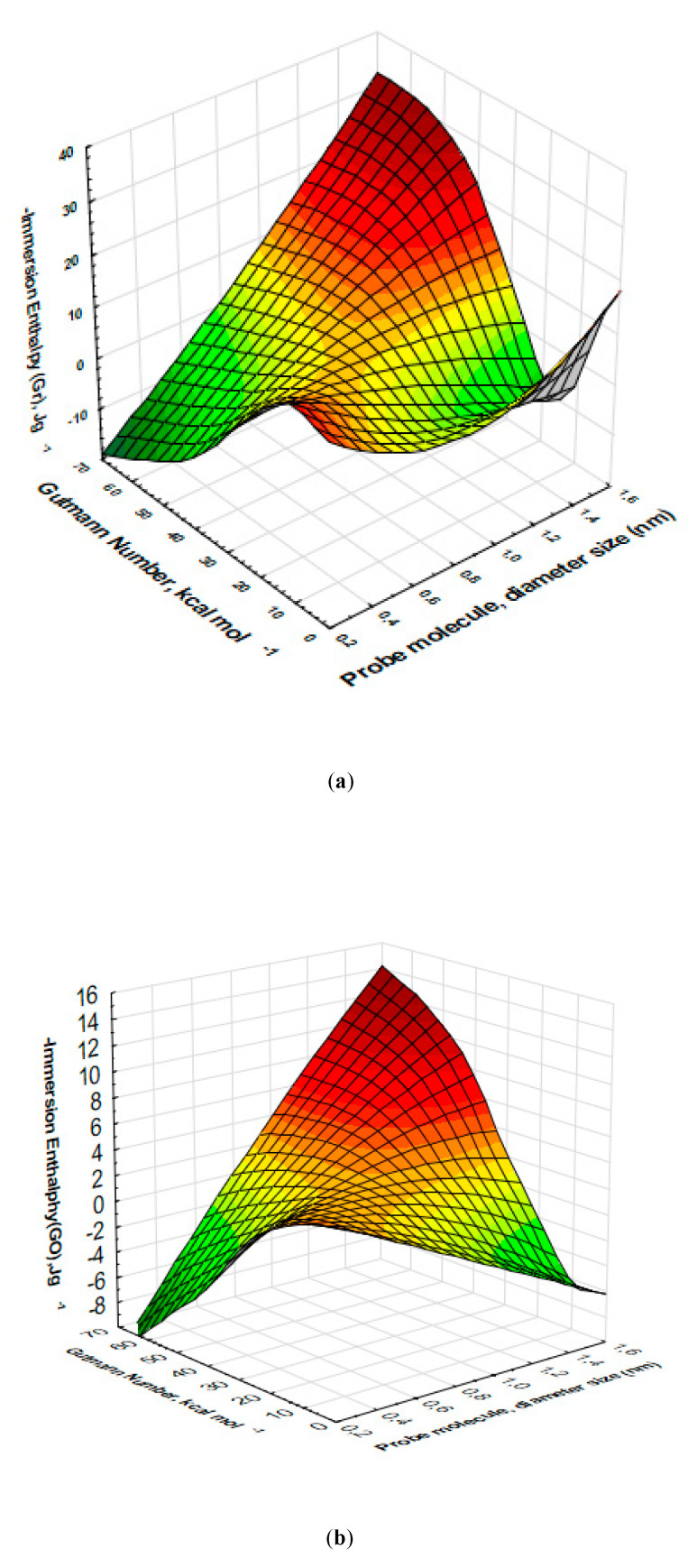

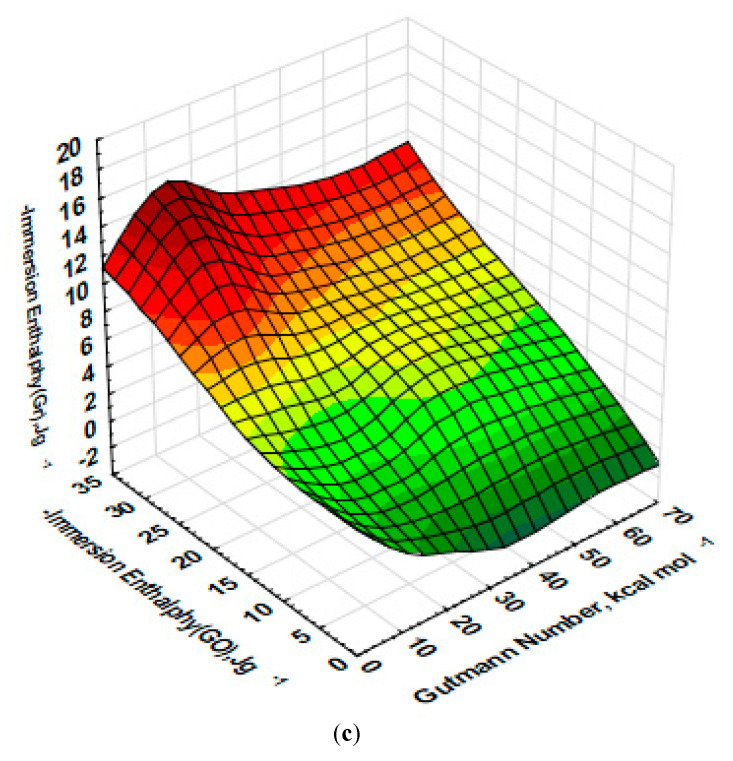
Surface representations: (**a**) immersion enthalpy-Diameter of probe molecule—DN Gutmann for Gr; (**b**) immersion enthalpy—diameter of probe molecule—DN Gutmann for GO; (**c**) immersion enthalpy—DN Gutmann Gr-GO.

Figure 5a (Gr) shows a graphic representation of the surface, between the Gutmann DN variables as a function of the diameter of the probe molecules and the immersion enthalpies. The result was very interesting: it observed that the enthalpic values were centered as a function of the Gutmann DN values between 20–40 kcal mol^−1^, which coincided with the behavior already analyzed between the diameter of the probe molecule and the immersion enthalpies. In Figure 5b (GO) the results were similar, the graph centered on values between 10–25 kcal mol^−1^ with respect to the Gutmann DN as a the enthalpies function corresponding to 1,3,5-Trimethybenzene (0.862 nm), Tri 2,4-xylyphosphate (1.50 nm) and ethanol (0.45 nm) that showed a defined centered band towards values that coincided with the PSD modeled with the QSDFT kernel.

Figure 5c shows various zones on the surface for both the Gr and the GO results that associated to the dispersive and specific components of the DN. Therefore, the graph taking into account the results of immersion calorimetry analyzed in three zones according to the characteristics of the solvents as follows: (i) that zone for the molecules with DN zero or very close to this value, it presented high values of immersion enthalpies. Among these molecules were hexane, cyclohexane, carbon tetrachloride, benzene, and dichloromethane.

The value obtained for these enthalpies was fundamentally associated with the effect of the interaction due to the entrance of the molecules into the pores of Gr and GO and their interaction with their walls. (ii) A second zone that could be observed in the surface graph (Figure 5c). Corresponded to those molecules whose Gutmann DN was between 10–20 kcal mol^−1^ with values of immersion enthalpies between 8.5 and 18.5 J g^−1^ corresponding to propanol and acetonitrile respectively for Gr, and enthalpic values between 27.0 and 32.0 J g^−1^ for i-propanol and ethanol molecules for GO. These values were higher compared to the previous ones and its was associated with interactions with functional groups, especially with those developed in GO, which were greater than those in Gr. (iii) Finally, a third zone was observed according to the Gutmann DN where the enthalpy values for both Gr and GO decreased again to values between 1.4 to 4.5 J g^−1^ for Gr, corresponding to molecules like Tri 2, 4-xylyphosphate and t-butanol and 3.5 and 9.0 J g^−1^ for Tri 2,4-xylyphosphate and t-butanol molecules for GO. These immersion enthalpy values were for DN between 25 and 61 kcal mol^−1^ and corresponded to Van der Waals interactions of those molecules that due to their size did not easily enter the GO porous structure. These results were consistent if the dielectric constant and the dipole moment were taken into account, values reported in Table 5. It was because these two constants were related to the Gutmann DN. If it was taken into account that the corresponding Gutmann DN was a quantitative measure of Lewis basicity, the results obtained in this investigation under our calorimetric experimental conditions were in good agreement, especially for GO, which was an acidic sample and it was the one that systematically presented the highest immersion heats. The results obtained by immersion calorimetry were also associated, for example, with polar and dispersive interaction effects for molecules such as water, ethanol, and 2-propanol, due to their polar character.

## 4. Conclusions

In this research, the PSD of the graphite (Gr) and the graphene oxide (GO) were studied in detail using the NLDFT and QSDFT kernels, modeling the slit pore geometry, since it was the one with the smallest percent error when these kernels were applied. To evaluate the PSD in this investigation, the microporous area was studied and it was compared with immersion enthalpies using different probe molecules. These molecules differed by their kinetic diameter, Gutmann’s DN, dipole moment, and dielectric constant. The results showed that the calorimetric technique is a useful tool to evaluate the PSD which presented comparable results to the homologous data deduced from the adsorption isotherms of N_2_ at 77 K using the QSDFT kernel, which was used in this work due to its best fit to the experimental data. The enthalpies of immersion showed magnitudes of immersion heats that were correlated with the Gutmann DN, which was additionally consistent with the acidity and basicity of solids determined by the Boehm method. This work showed that the calorimetry allowed to track the PSD in a more realistic way without making approximations as other mathematical models did.

## Figures and Tables

**Table 1:** Characteristics of the liquids used in the determination of the enthalpy immersion.

Solvent	CAS No.	Source	Initial ^a^ Purity	Purification Method	pKa
Benzene	71-43-2	Sigma Aldrich	≥99.9%	HPLC	43
Dichloromethane	75-09-2	Sigma Aldrich	≥99.9%	HPLC	-
Hexane	110-54-3	Sigma Aldrich	≥97.0%	HPLC	50
Acetonitrile	75-05-8	Sigma Aldrich	≥99.9%	HPLC	25
Triethylamine	121-44-8	Sigma Aldrich	≥99.5%	GC	10.8
Water	7732-18-5	Sigma Aldrich	Water plus	supplier	15.7
Ethanol	64-17-5	Sigma Aldrich	95%	supplier	16
Propanol	71-31-8	Sigma Aldrich	99.7%	supplier	16.1
i-Propanol	67-63-0	Sigma Aldrich	95.0%	supplier	17.1
Cyclohexane	110-82-7	Sigma Aldrich	95.0%	supplier	45
Carbon Tetrachloride	56-23-5	Sigma Aldrich	99.9%	supplier	-
t-Butanol	75-65-0	Sigma Aldrich	≥99.5%	HPLC	19.20
p-Xylene	106-42-3	Sigma Aldrich	≥99.0%	GC	15
1,3,5-trimethylbenzene	108-67-8	Sigma Aldrich	95.0%	supplier	14
Tri-2,4-xylyphosphate	31570-04-4	Sigma Aldrich	98.0%	supplier	-

^a^ Data supplied by the company.

**Table 2:** DR and DFT parameters obtained from the adsorption-desorption isotherms of N_2_ at −196 °C [86].

Samples	S_BET_ [m^2^ g^−1^]	DR (P/P^o^ < 0.1)				DFT (P/P^o^ 10^−7^)	
		V_mic_ [cm^3^ g^−1^]	E_o_ [kJ mol^−1^]	n	Pore Radius [Å]	V_p_ [cm^3^ g^−1^]	Half Pore Width [nm]
Graphite (Gr)	5.2	0.010	7.250	3.4	7.4	0.04	7.80
Graphene (GO)	47.5	0.154	18.60	5.4	9.3	0.18	8.75

**Table 3:** Fitting error for different surface textures (NLDFT vs. QSDFT) in slit and slit/cylindrical pores [86].

	NLDFT			QSDFT		
Sample	Fitting Error (slit pore) [%]	Fitting Error (cyl. pore) [%]	Fitting Error (cyl.-slit) [%]	Fitting Error (slit pore) [%]	Fitting Error (cyl. pore) [%]	Fitting Error (cyl.-silt) [%]
Graphite (Gr)	5.296	6.819	6.819	4.364	7.510	7.910
Graphene O_x_. (GO)	2.345	3.576	4.765	1.354	2.546	3.087

**Table 4:** Superficial groups (μmol g^−1^) for Gr and GO determined by the Boehm method.

	Groups	Lactonic μmol g^−1^	Carboxylic μmol g^−1^	Phenolic μmol g^−1^	Carbonyl μmol g^−1^	Total Acidity μmol g^−1^	Total Basicity μmol g^−1^	Total Groups μmol g^−1^
Samples								
**Gr**		0.000 *	0.000 *	0.000 *	0.000 *	0.000 *	0.000 *	0.000 *
**GO**		1.282 ± 0.003	0.626 ± 0.005	0.023 ± 0.0001	0.025 ± 0.004	3.564 ± 0.007	1.639 ± 0.006	5.203 ± 0.008

* BD = below detection limit.

**Table 5:** Characteristics of the probe molecules used in the calorimetric study.

Solvent	Molecular Size (nm)	Gutmann Number (kcal/mol)	Structure	Dipole Moment	Dielectric Constant
Benzene	0.585	0.1	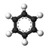	0	2.28
Dichloromethane	0.334	1	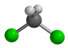	1.58	9.1
Hexane	0.492	0	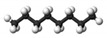	0	2.0
Acetonitrile	0.272	14.1	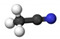	3.84	37
Triethylamine	0.770	61	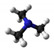	1.60	2.42
Water	0.293	18	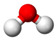	1.85	80.1
Ethanol	0.450	19.2	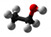	1.77	24.5
Propanol	0.490	19.8	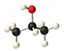	1.68	
i-Propanol	0.472	21.1	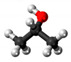	1.56	17.9
Cyclohexane	0.482	0.0	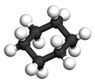	0	2.02
Carbon Tetrachloride	0.595	0.0	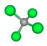	0	2.24
t-Butanol	0.605	38.0	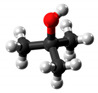	1.70	15.8
p-Xylene	0.665	5.0	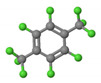	0.02	2.27
1,3,5-trimethylbenzene	0.862	10.0	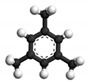	0.10	2.40
Tri-2,4-xylyphosphate	1.50	25.0		1.5	25.0
